# Multi-analytical study of the medieval wall paintings from the rupestrian church *Grotta del Crocifisso* at Lentini (eastern Sicily): new evidence of the use of woad (*Isatis tinctoria*)

**DOI:** 10.1007/s12520-022-01656-6

**Published:** 2022-09-03

**Authors:** Giuseppe Montana, Renato Giarrusso, Raffaella D’Amico, Barbara Di Natale, Mirko Andrea Vizzini, Vincenzo Ilardi, Angelo Mulone, Luciana Randazzo, Claudio Ventura Bordenca

**Affiliations:** 1 Dipartimento di Scienze della Terra e del Mare (DiSTeM), Università degli Studi di Palermo, Palermo, Italy; 2 GEOLAB s.r.l Laboratorio di Ricerca e Sperimentazione sui Materiali, Via de Spuches, Carini, Palermo, Italy; 3 Conservator-Restorer, Parco Archeologico di Leontinoi (Regione Siciliana), Siracusa, Lentini Italy; 4 Restorer, Palermo, Italy; 5 Dipartimento di Scienze e Tecnologie Biologiche Chimiche e Farmaceutiche (STEBICEF), Università degli Studi di Palermo, Palermo, Italy

**Keywords:** Middle ages, Sicily, Wall paintings, Woad (*Isatis tinctoria*), Thin-section microscopy, p-XRF, SEM-EDS, FTIR, Raman spectroscopy

## Abstract

This study presents the results of the examination and characterisation of the wall paintings that decorate the rupestrian church named *Grotta del Crocifisso*, which is located in the territory of Lentini (eastern Sicily, few tens of kilometres from Catania and Syracuse). The earliest mural paintings in the church date back to the twelfth century AD. A multi-analytical approach was adopted for the characterisation of stone materials, secondary degradation products, and pigments. For this purpose, the following techniques were used: reflected light microscopy (RLM), polarised light microscopy on thin sections (PLM), X-ray powder diffraction (XRPD), mercury intrusion porosimetry (MIP), portable X-ray fluorescence (p-XRF), scanning electron microscopy (SEM-EDS), Fourier-transform infrared spectroscopy (FTIR), and Raman spectroscopy (RS). The lithic substrate and the plaster’s coating layers were thoroughly characterised from compositional and textural points of view, and the use of locally available raw materials was established. Similarly, the newly formed crystalline phases produced by alteration processes of the original materials were recognised. The red, yellow, brown, and green pigments were easily identified by p-XRF and SEM-EDS. The use of “earth pigments” widely available in the surrounding area (various types of ochre) was thus highlighted. The recognition of the dark blue pigment created some additional issues in its identification, making further diagnostic methods necessary. In fact, the use of the most common mineral pigments was categorically excluded by both p-XRF and SEM-EDS, since no chromophore metallic elements were highlighted with the exception of trace amounts of iron. A combination of detailed microscopic observations together with the application of FTIR and RS supported the use of an organic pigment obtained from the maceration of woad (*Isatis tinctoria*). The green pigment is the result of a mixture between woad and yellow ochre. Woad is even today easily available in Sicily, and some additional experimental tests were carried out on *Isatis tinctoria* that had been freshly collected in the area (treated with traditional procedures). Over the past centuries, woad was widely used for dyeing fabrics, but its practice for wall paintings has only been sporadically proven. The identification reported in this case study could be considered a novelty at least in the Sicilian panorama.

**Supplementary Information:**

The online version contains supplementary material available at 10.1007/s12520-022-01656-6.

## Historical background and aims

**Fig. 1:**
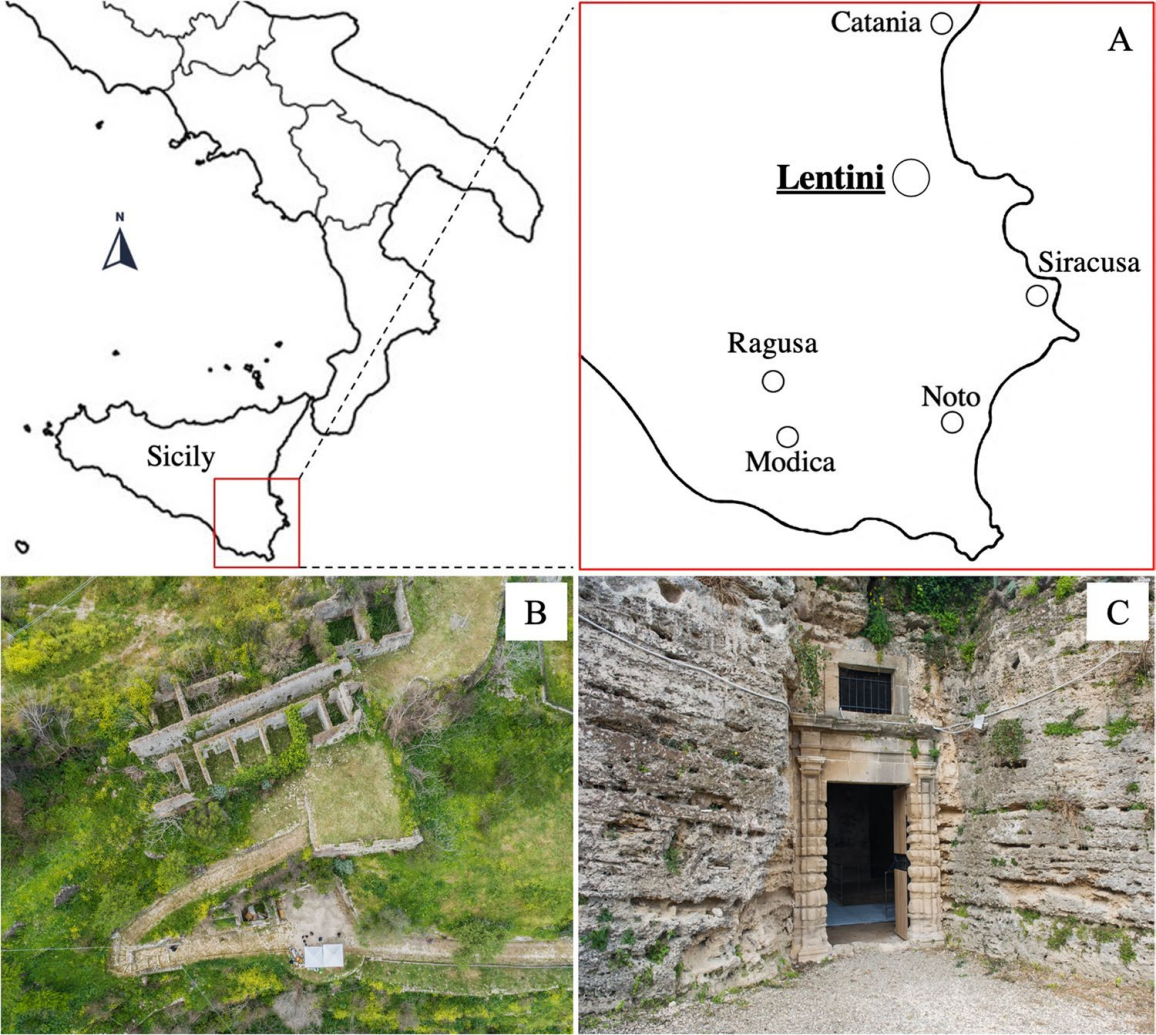
(A) Location of the Grotta del Crocifisso in eastern Sicily; (B) aerial view of the site; (C) main entrance built in 1764

**Fig. 2:**
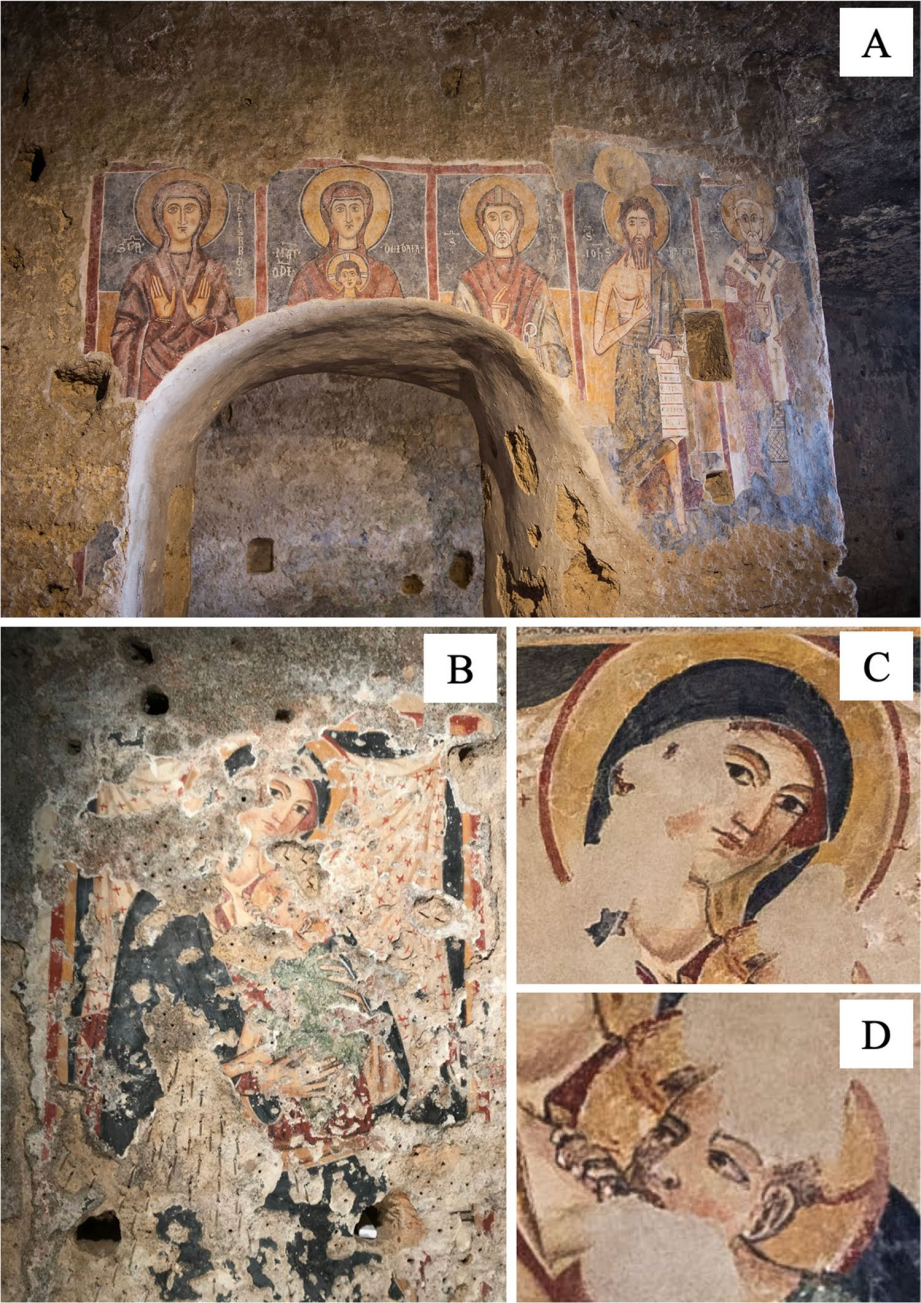
(A) Overview of the complete Teoria dei Santi after the restoration work; (B) overview of the Madonna del Latte before the restoration work; (C) and (D) details of the faces of the Madonna and Child Jesus after the restoration work

The rupestrian church *Grotta del Crocifisso* is located in the Archaeological Park of *Leontìnoi* in the territory of the modern city of Lentini, a few tens of kilometres from Catania and Syracuse (DMS coordinates: 37°16′ 49″ N, 15° 00′ 29″ E). The site is at the northern border of the Hyblean Mountains, in south-eastern Sicily (Fig. 1). It held a strategic position in very ancient times providing an important connection along the south-north road axis and the interior of the island. Since its discovery by Paolo Orsi in 1917, the church has been the subject of various historical-artistic studies (e.g. Messina 1979). The rupestrian church is dedicated to the *Madonna della Grutta*, as evidenced by the archival documents and the decorative theme represented on the northern wall and houses a cycle of frescoes dating back between the twelfth and seventeenth centuries (Messina 1979). The wall paintings of the sacred area and (partly) of the two innermost rooms constitute the greatest wealth of this rupestrian church which, due to its rich cycle of frescoes, is considered the most important in the whole of Sicily. They are an expression of specialised local artists that possess a pictorial repertoire of which many examples, albeit fragmentary, still remain in eastern Sicily, i.e. paintings of the crypt of *San Marziano* and those of the oratory of *Santa Lucia* in Syracuse, the frescoes of the Cave of *Santa Lucia* and *Santa Margherita* in Lentini, and the Cave of the *Santi delle Pianette* in Palazzolo Acreide and those of Pantalica in the territory of Sortino (Arcidiacono 2019). The typology of these churches, both from an architectural and pictorial point of view, arises from the need for a rechristianisation of the island desired by the Normans which draws on a Byzantine iconographic repertoire and which differs from area to area through a relatively complex articulation. The iconographic system of the *Grotta del Crocifisso* consists of five decorative phases that follow one another and overlap each other, as up to three layers of frescoes, where often the iconographic themes of the oldest frescoes are often repeated in later ones. The most ancient phases dating back to the end of the twelfth century depict a repertoire, which has its roots in the Byzantine world, both in iconographic and iconological value, retracing the main stages in the history of Salvation. The frescoes of the second phase, dating to the thirteenth century, provide evidence of an iconographic repertoire whose matrix is attributable to the territories of Jerusalem and Palestine, from where the emigration of monastic and military orders took place, in particular that of the Templars (Campagna Cicala 2020). The major pictorial remains of the *Grotta del Crocifisso* are testimonials of this latter phase. The frescoes, object of this diagnostic study, were the polyptych representing the *Teoria dei Santi* (Theory of Saints), dated to the thirteenth century, depicting *Santa Elisabetta*, a *Madonna Odigidria*, *San Leonardo*, *San Giovanni Battista*, and *San Nicola* (Fig. 2A). The panel dedicated to the *Madonna del Latte*, dated between the end of the fourteenth and the beginning of the fifteenth centuries, was also studied (Fig. 2B–D).

The structure of the *Grotta del Crocifisso* has undergone to various transformations over the centuries (Messina 1979). Today, it consists of two square-shaped rooms, with similar dimensions, which are flanked and connected by a small corridor (ESM 2A). The left room was used to welcome the faithful during the celebration of religious rites. The adjacent room, with its own entrance, was the prerogative of the priests. Here the altar, entirely carved into the rock, with a characteristic ogive-shaped apsidal basin placed in the centre of the wall, faces eastward as in the Syro-Palestinian ecclesiastical systems of the Orthodox tradition (Messina 1979). Over the centuries, the cave was enlarged through the creation of two more internal rooms and two hypogea with a barrel vault which were used for burials. One of the latter is placed beneath the area dedicated to the faithful followers and has gully seats carved into the rock. The convent of hermit monks above the cave, probably dating back to the seventeenth century, and the two rooms to the south used as ossuaries, also date back to this period. Subsequently, the west room at the bottom of the cave was enlarged, and the second hypogeum was built. The last substantial renovations to the structure date back to 1764; the date is inscribed on today’s entrance portal, with the opening of two large arches that partially destroyed the frescoes created in correspondence with the walls that separated the rooms.

The whole decorative complex was in a worrying state of decay in 2017, when the planning of the first restoration intervention was decided on. Most of the previously cited pictorial representations were clouded by widespread coherent saline encrustations, efflorescences, biological patinas (Cyanophytic algae) and also affected by the fragmentation and detachment of considerable portions of the substrate (ESM 2B). From the first inspection, the dissolution-precipitation cycles of soluble salts have been recognised as the most relevant cause of deterioration, involving both the pictorial films and the plaster layer. In order to characterise all the categories of stone materials involved in this decorative complex (bedrock, mortar/plaster layers, finishings, pigments) and the degradation products (encrustations and efflorescences), a series of analytical diagnostic investigations were planned, including: reflected light microscopy (RLM), polarised light microscopy on thin sections (PLM), X-ray powder diffraction (XRPD), mercury intrusion porosimetry (MIP), portable X-ray fluorescence (p-XRF), scanning electron microscopy (SEM-EDS), Fourier-transform infrared spectroscopy (FTIR), and Raman spectroscopy (RS). A basic study of the cave microclimate was been also carried out and is ongoing today, through measurements of temperature (T) and relative humidity (RH%), both fundamental parameters for restoration and conservation aims. The microclimate should first be acknowledged and then stabilised, in order to preserve the works of art. In addition to the characterisation of the used materials, this analytical approach was primarily aimed at providing guidance for the implementation of restorative intervention strategies and insights into the nature and provenance of the raw materials and manufacturing procedures.

## Sampling and analytical methods

The sampling (methods, sample number, sample size, etc.) and selection of the analytical procedures/techniques are considered central steps for any scientific work in the field of Cultural Heritage (Artioli 2010).

In the case study addressed in this paper, the first points of interest are the calcareous sandstone composing the substrate of the *Grotta del Crocifisso* and the layers of lime mortars which, on the whole, were used to prepare the walls for decorative paintings. Similar attention was also paid to the characterisation of the pigments used in the painted walls, essentially represented by yellow, red, green, brown, and blue. On this basis, after a thorough in situ visual examination, a set of representative samples was accurately collected. Specifically, the selection of plaster samples was inductively limited to the painted panel of the *Madonna del Latte* and of the polyptych *Teoria dei Santi*, which, in turn, were selected as priority sectors for restoration works. A list of the studied samples their nature, collection points, and the analytical methods used for their characterisation is provided in the Supplementary Materials (ESM 1). The laboratory investigations were carried out at the DiSTeM (University of Palermo), at GEOLAB (Carini, Italy), and at the DiBEST (University of Calabria):

- Polarising light microscopy (PLM) was performed on polished thin sections. The latter were first observed under a reflected light microscope at relatively low magnification (stratigraphy, mesostructure) and subsequently under transmitted light with a polarising microscope Leica DM-SLP, equipped with a digital camera (DiSTeM laboratory).
- X-ray powder diffraction (XRPD) was carried out with a PANanalytical X’Pert Pro diffractometer (Philips Analytical model), equipped with a graphite monochromator crystal. The measuring conditions were as follows: CuKα radiation, potential 40 kV, filament current 40 mA, and goniometer speed 2°2θ/min (DiSTeM laboratory).
- Scanning electron microscopy (SEM-EDS) under low vacuum conditions (i.e. without carbon or gold sputter coating) was carried out on tiny portions of the small samples detached from the masonry (fresh fracture surface). Observations and qualitative compositional analyses were made with a SEM/ESEM model FEI Quanta 200, equipped with an EDAX microanalysis system. The operating conditions were as follows: 20 kV accelerating voltage, 1.2 nA beam current, working distance equal to 10 or 20 mm, and counting times up to 100 s (GEOLAB laboratory).
- A non-invasive in situ investigation by portable X-ray fluorescence (p-XRF) was performed with an AMETEK spectrometer consisting of a Mini-X2 X-ray tube equipped with an Au anode and a SDD solid-state silicon drift detector with a Be window and a typical resolution of 125 eV. Analytical measurements were performed with a voltage of 40 kV and current of 15 μA, working distance of about 90 mm from the sample, and acquisition times of 150 s (GEOLAB laboratory).
- Fourier-transform infrared spectroscopy analysis (FTIR) was performed only on dark blue pigment samples with a Bruker Tensor 27 spectrophotometer equipped with an attenuated total reflectance (ATR) accessory. Infrared spectra were recorded in ATR mode, in the range of 500–4,000 cm^−1^, producing 32 scans at a resolution of 4 cm^−1^ directly obtained from the external surface of the sample (DiSTeM laboratory).
- Raman spectroscopy (RS) measurements (performed only on the dark blue pigment samples) were conducted using a confocal Raman microscope (NRS 5100-Series Raman, from Jasco) equipped with a SHG Nd:YAG laser (532 nm) and a lens-based spectrometer and using 1800 mm^−1^ diffraction gratings (DiBEST laboratory). The spectral resolutions were 4.18 cm^−1^ and 1.09 cm^−1^/pixel. Spectra were recorded at selected positions with an integration time of 30 s and 3-fold averaging at 6.0 mW. A 100× microscope objective was used for the measurements. Two or three spots were analysed on each sample. The spectra were collected in the range of 500–1800 cm^−1^. The Raman spectra were exported to a ASCII format and subsequently analysed using OriginPro, Version 2021 for baseline correction (OriginLab Corporation, Northampton, MA, USA).
- Mercury intrusion porosimetry (MIP) analysis was performed on the lithic substrate as well as on representative mortar samples (supporting and rendering layers) for determining pore-size distributions. A Micromeritics Autopore IV mercury porosimeter (pressure range 25 kPa up to 400 MPa, pore-size range 40–0.003 μm) was used for this purpose (DiBEST laboratories).
- The measurements of the microclimatic parameters (T and RH) were carried out in three different sectors of the *Grotta del Crocifisso*. The sensors were positioned at a minimum height of 50 cm from the ground, and the data were stored with EBI 300 data loggers equipped with TPH 400 temperature and humidity probes (accuracy ± 0.5 ° C T and ± 3% RH).

## Results

### Lithic substrate

#### Geological context

**Fig. 3:**
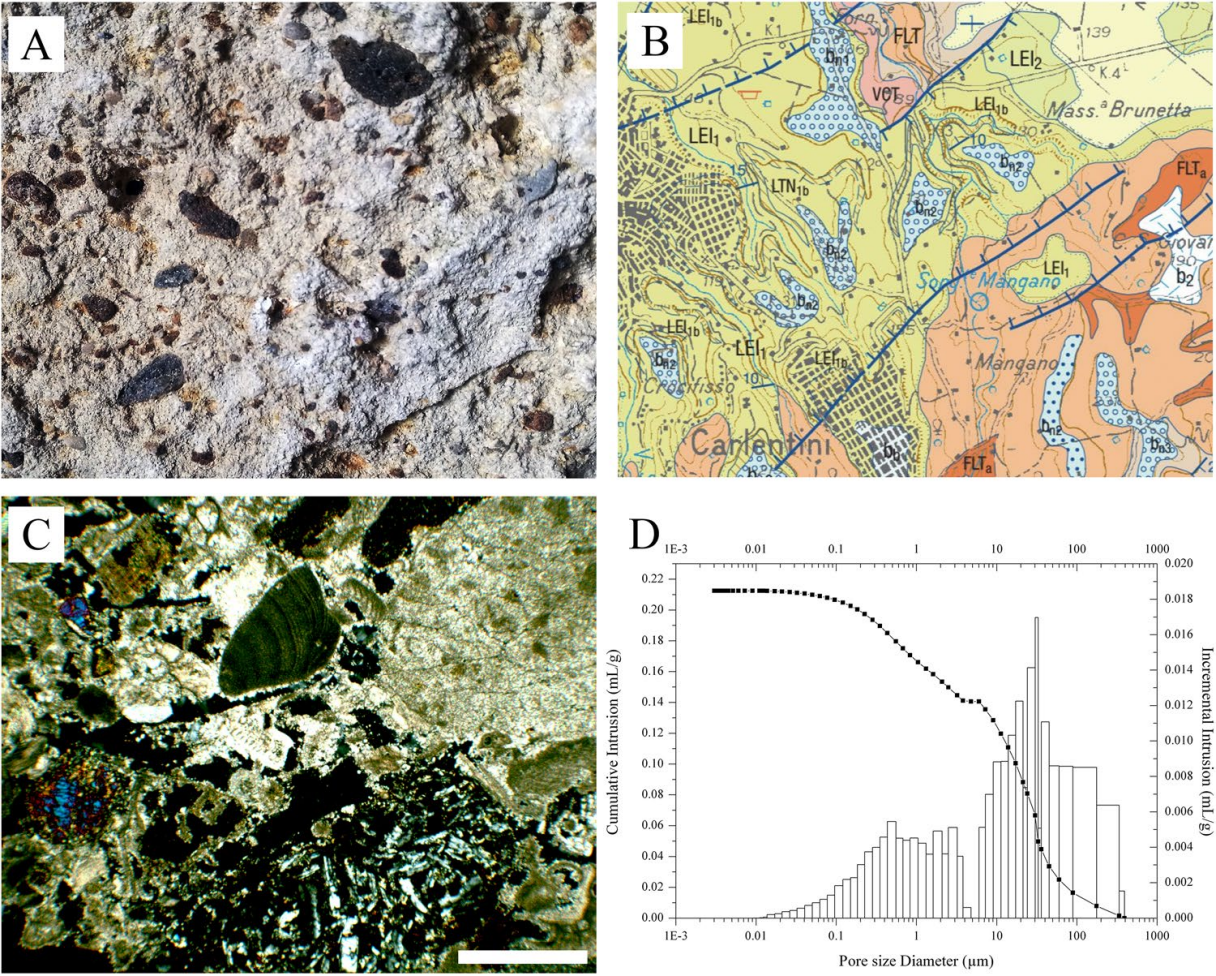
(A) Macroscopic image of the local calcarenite at the entrance of the hypogeum showing the presence of volcanic clasts; (B) geological map from Progetto CARG (Carta Geologica d’Italia scala 1:50.000) Foglio 641 Augusta(Carbone, 2011). Legend: b2 = eluvial-colluvial deposits (Holocene); bb = recent alluvial deposits (Holocene); bn1-3 = terraced alluvial deposits (Late Pleistocene); LEI1 and LEI1b = fossiliferous yellowish calcarenites and sands (Villasmundo subsynthem; Lower Pleistocene); LEI2 = grey-blue clays and silty-marly clays (Scordia subsynthem; Lower Pleistocene); VCT = tholeitic basalts (Militello in Val di Catania Formation; Middle-Late Pliocene); FLT and FLTa = volcanoclastites with abundant sedimentary carbonates and subordinate Na-alkaline basaltic flows (Carlentini Formation; Upper Miocene); (C) Thin section microphotograph of the lithic calcarenite substrate (sample CC-1), in cross-polarised light (XPL), scale bar = 0.5 mm; (D) porosimetric analysis of the lithic substrate showing a polymodal pore-size distribution

The *Grotta del Crocifisso* rupestrian church has been carved in a light-yellow, medium-coarse to coarse-grained fossiliferous calcarenite (or biocalcarenite) characterised by the remains of calcareous macrofauna (ostreids, pectinids, corals) and black to reddish-brown heterometric (up to 2–3 cm) volcanic lithoclasts (Fig. 3A). The hypogeum lithotype belongs to the Lentini Synthem (LEI) of lower Pleistocene age. The Lentini Synthem consists of marine sediments with various textures and depositional environments grouped in the subsynthems of Villasmundo (LEI_1_) and Scordia (LEI_2_) showing latero-vertical heteropic relationships (Fig. 3B). The first subsynthem (Villasmundo) includes a predominantly calcarenite-sandy succession together with a conglomeratic lithofacies, mostly in the basal position. The second subsynthem (Scordia) is represented by a clayey succession topped by sand-silt lenses. Calcarenite and/or sand lithologies define a proximal marine sedimentation environment, while the clay lithologies delineate the central depositional zones of tectonic depressions. The synthemic unit is demarcated below by an ENE-verging angular unconformity on a substrate consisting of Upper Cretaceous-Lower Pleistocene limestones and volcanic rocks.

#### Minero-petrographic characterisation

Based on the petrographic examination under the polarising microscope, the lithic substrate can be classified as a grainstone (after Dunham 1962) with variable amounts of matrix (microcrystalline calcite) and microsparitic cement. It is characterised by a prevalent calcareous bioclasts, represented predominantly by abundant benthic foraminifera and minor amounts of bryozoans, calcareous algae, mollusks, and fragments of echinoids (spicules). Ostracods and planktonic foraminifera are sporadic to rare. A not negligible volcaniclastic component is also present, consisting of both lithic fragments and monomineralic granules which fully reflect the composition of the volcanic products of the Hyblean plateau (hyaloclastites, breccias, and pillow lavas mainly with tholeitic compositions). Among the lithic fragments (the coarser grains of the sandy fraction), basalts with a glassy groundmass with plagioclase and clinopyroxene phenocrysts (either as single crystals or mixed with the carbonate component) are prevalent (Fig. 3C). Angular fragments of sideromelane (basaltic glass) are also common, sometimes in an incipient state of palagonitisation.

The XRPD patterns corroborated the results concerning the mineralogical composition achieved by means of polarised light microscopy. Calcite is the most abundant mineralogical phase, while clinopyroxene and plagioclase are relatively subordinate. The MIP analysis allowed the evaluation of the total connected porosity, volume of pores, and pore-size distributions between 0.001 and 300 μm. As shown in Fig. 3D, the pore-size distribution frequencies tend to assume a polymodal to bimodal trend. The classes of macropores with diameters between 10 and 80 μm are visibly prevalent. Nevertheless, macropores with a relatively smaller diameter (0.1–1 μm) and, subordinately, mesopores with diameters between 0.01 and 0.05 μm are also represented.

### Plasters

#### Stratigraphy and macroscopic description

**Fig. 4:**
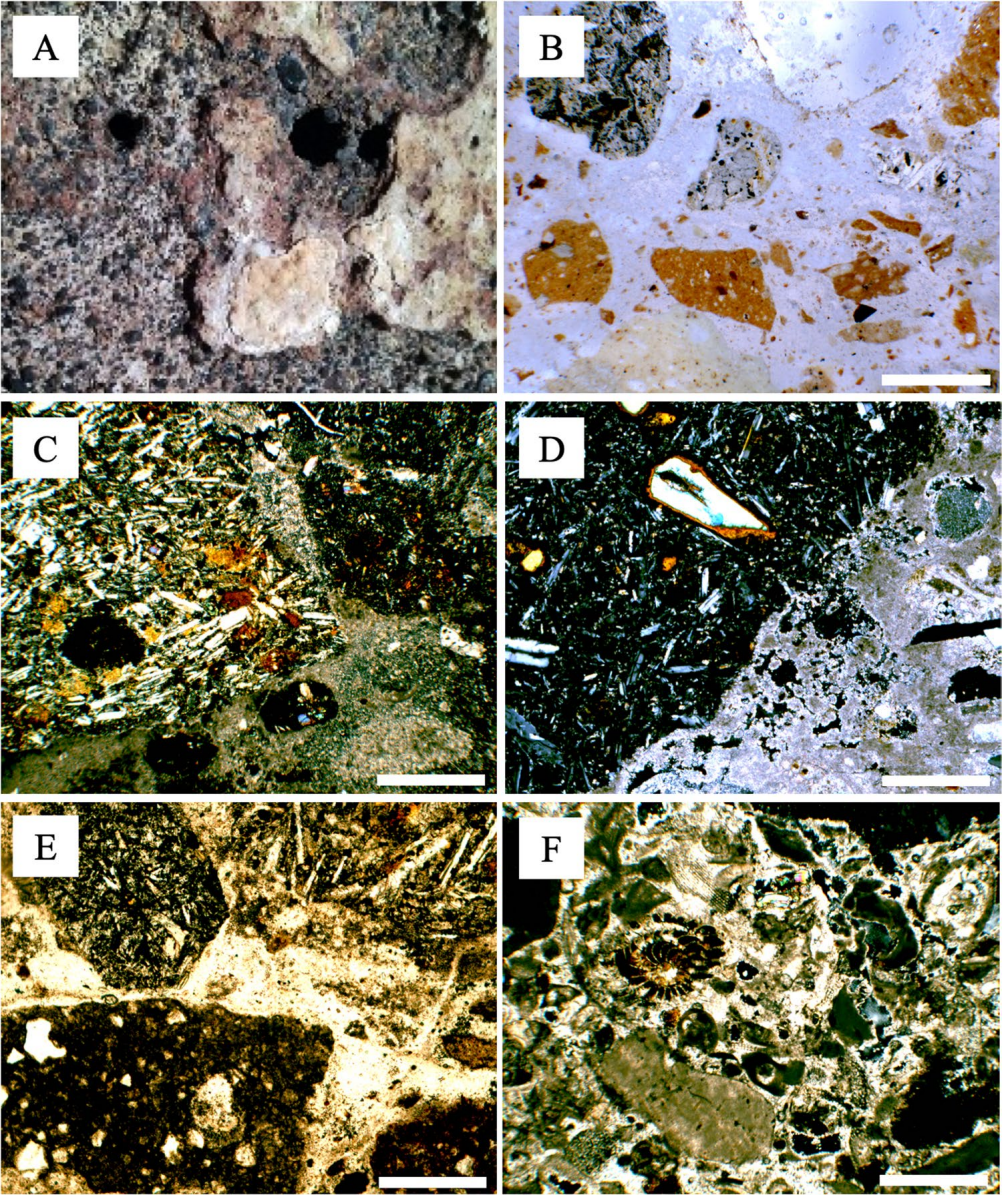
(A) Macroscopic picture of the plaster layers; (B) reflected light image showing the cocciopesto fragments (sample CR/AR-2); (C) thin section microphotograph of the greyish arriccio layer (sample CR/AR-1) showing volcanic clasts, in cross-polarised light (XPL); (D) thin section microphotograph showing the reaction rim between the binder and aggregate in the greyish arriccio layer (sample CR/RN-2), XPL; (E) thin section microphotograph of the pinkish arriccio layer (sample CR/RN-1) showing cocciopesto and volcanic clasts, in plane-polarised light (PPL); (F) thin section microphotograph of the tonachino layer (sample CR/FC-1) showing bioclasts, in cross-polarised light (XPL). Scale bar = 0.5 mm

The plasters that sustain the wall paintings of the *Grotta del Crocifisso* showed a typical stratigraphy. Two layers with different functions, thicknesses, and compositional characteristics can be distinguished starting from the calcarenite substrate. Foremost, there is a greyish and very coarse-grained layer set directly in contact with the stone. This layer, which can be defined as *arriccio*, clings to the underlying stone, filling and levelling the wall in its roughness for the subsequent drawing of the fresco. In the relatively more recent frescoed walls (manufactured starting from the fourteenth century), the *arriccio* level has been replaced in its function by a mortar level with a similar aggregate size (even if slightly finer) clearly recognisable by its pinkish colour. Both these layers are covered by the same whitish and medium-fine grained finish coating layer (*tonachino*) directly supporting the fresco paintings. This representative stratigraphic succession can be discernibly attested in several points in the wall where a mortar cover is lacking, due to degradation processes (Fig. 4A). The above-described layers of plaster show different macroscopic textural characteristics. The greyish *arriccio* layer is characterised by variable thickness (up to a several centimetres) and by a very coarse aggregate consisting of dark lithic volcanic fragments. The pinkish *arriccio* layer is characterised by clearly visible *cocciopesto* (crushed terracotta) and volcanic fragments (Fig. 4B). The finish coating layer (*tonachino*) is whitish in colour and shows a smaller thickness than the underlying layers (some millimetres). In this layer, calcareous grains predominate in the aggregate and volcanic fragments are rare or even absent.

#### Minero-petrographic characterisation

The investigations carried out under the polarising microscope on the greyish *arriccio* confirmed the use of an aggregate consisting almost exclusively of basaltic rock fragments ascribed to local outcrops. The observed lithic fragments are characterised by variable textures ranging from holocrystalline intersertal or intergranular to hypocrystalline or vitrophyric, with phenocrysts of plagioclase, clinopyroxene, olivine, and opaque oxides (Fig. 4C). Angular fragments of sideromelane (basaltic glass) are also common, sometimes being in an incipient state of palagonitisation (formation of palagonite by the hydration of volcanic glass). Femic minerals sometimes appear intensely altered to iron oxides. The grain size ranges from coarse sand (0.5–1 mm) up to very coarse sand (1–2 mm) and very fine gravel (mostly 2–8 mm). The aggregate/binder ratio, obtained from the assessment of aggregate grain packing using standard comparison charts, varies from 2:1 to 3:1 (Matthew et al. 1991). The binder matrix appears optically isotropic, being mostly represented by a colloform mass of hydrated calcium silicates of amorphous nature (CSH) formed after the “pozzolanic reaction” between the original aerial lime and the highly reactive volcanic glass composing the aggregate. The binder also displays sporadic patches of microcrystalline calcite, resulting from hydrolysis reactions and which are mostly developed along the primary cracks of the amorphous mass or in contact with the aggregate granules (Fig. 4D). Condensation moisture penetrates into the plaster, and upon contact with H_2_O and atmospheric CO_2_, the CSH are retrogressively altered into microcrystalline calcite. Moreover, in some areas, the “pozzolanic reaction” seems to be less severe and the binder appears largely carbonated as evidenced by patches of microcrystalline calcite resulting from the primary carbonation of the aerial lime binder. Such a heterogeneity concerning the degree of “pozzolanic reaction” in the binder may be caused both by the random predominance of volcanic clasts with holocrystalline structures (with absent or very weak pozzolanic reactivity) in the aggregate and by the local variations of the thermo-hygrometric conditions during the curing phase.

The pinkish *arriccio*, similarly to the previous one, is characterised by the presence of volcaniclastic aggregates, consisting of sub-rounded granules mainly falling into the medium sand class (0.25–0.5 mm) and having a size of up to 2 mm. The rock fragments were recognised as basalts with holocrystalline and hypocrystalline or vitrophyric (sideromelane) textures. The mineral paragenesis is defined by plagioclase, olivine, and clinopyroxene, which are often strongly oxidised to haematite (Fe_2_O_3_) and goethite (FeOOH). Moreover, fragments of *cocciopesto* (variably sized up to very coarse sand) were identified as common constituents in this layer (Fig. 4E). Microscopic observations showed that the co-presence of *cocciopesto* and oxidised volcanoclasts gives the plaster an overall pale reddish colour. The binder groundmass appears optically isotropic with no birefringence and is opaque if observed under plane-polarised light. The “pozzolanic reaction” between volcanic glass (aggregate) and the aerial lime binder formed CSH, which are notoriously non-crystalline and thus non-birefringent phases. As in the previously described case of the greyish arriccio-rendering layer, various patches of microcrystalline or microsparitic calcite resulting from the retrograde hydrolytic reaction in the pozzolanic binder can be observed.

The *tonachino* layer differs from both the previously described (underlying) *arriccio* layers, showing an aggregate consisting only of calcareous bioclasts and fragments (from angular to subangular) obtained after grinding local calcarenite (Fig. 4F). The grain-size distribution of the aggregate is relatively more sorted, with a prevalence of medium-fine sand (0.2–0.4 mm) over coarse sand (1–2 mm). The aggregate/binder ratio was estimated to be 1:4 (around 20% area). Unlike that of the underlying layers, the binder matrix is entirely made up of carbonated lime. The texture varies from microcrystalline to cryptocrystalline in irregular patches, with a tendentially heterogeneous structure and numerous lumps. Pervasive primary shrinkage, as a consequence of air trapping, is evident from the presence of cracks and voids.

**Fig. 5:**
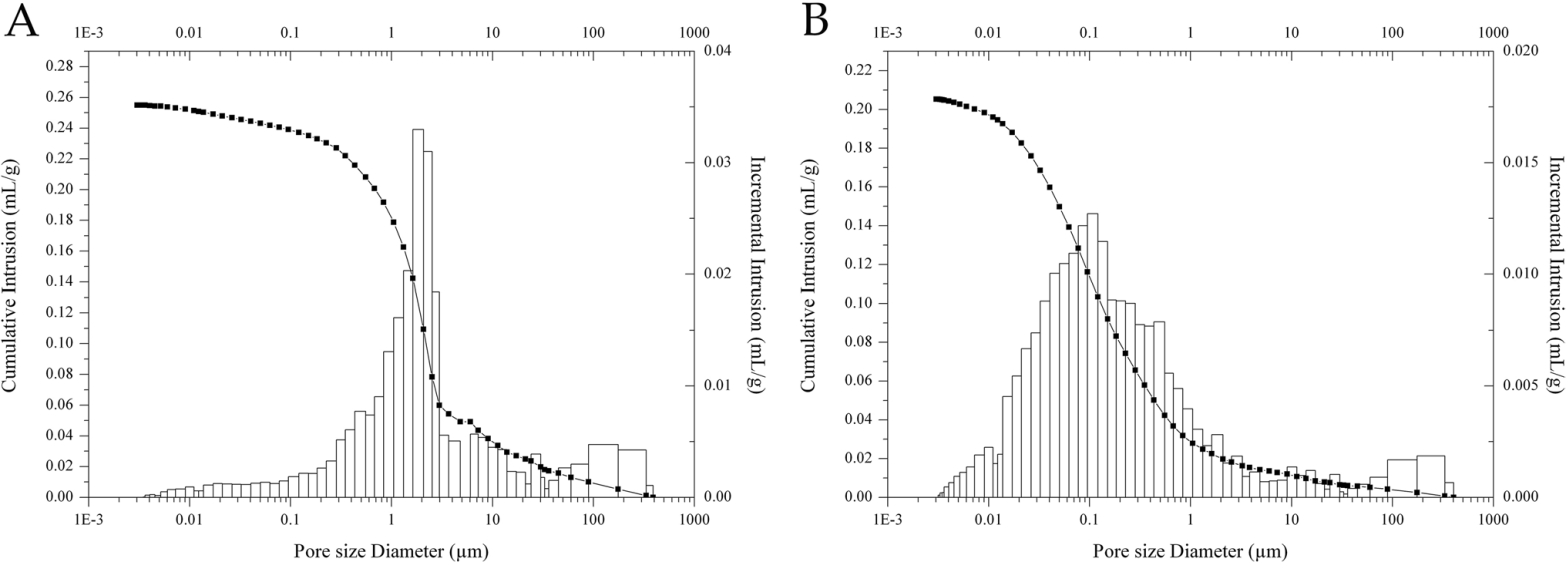
Porosimetric analysis of the (A) arriccio layer and (B) tonachino layer

The MIP analysis of the supporting plaster for wall paintings is shown in Fig. 5. The pore-size distribution frequency of the *arriccio* layer (Fig. 5A) is clearly distinguished by the prevalence of small macropores with diameters between 1 and 3 μm, although macropores with greater (10–100 μm) or smaller (0.01–1 μm) diameters are not negligible. On the contrary, the *tonachino* layer shows higher frequencies of the relatively larger mesopores (0.01–0.05 μm) and smaller macropore (0.05–0.5 μm) classes, while macropores with diameters > 0.5 μm are less represented in this layer (Fig. 5B).

#### Alteration and degradation products

The newly formed compounds deriving from the alteration and degradation processes of the plaster (and to a lesser extent also of the calcarenite substrate) were characterised by means of the XRPD and SEM-EDS techniques. In the wall paintings of the *Grotta del Crocifisso*, two types of secondary products were recognised as having formed in this particular environment over the centuries: encrusting deposits and efflorescences of soluble salts. The latter were also found developed directly on the calcarenite.

**Fig. 6:**
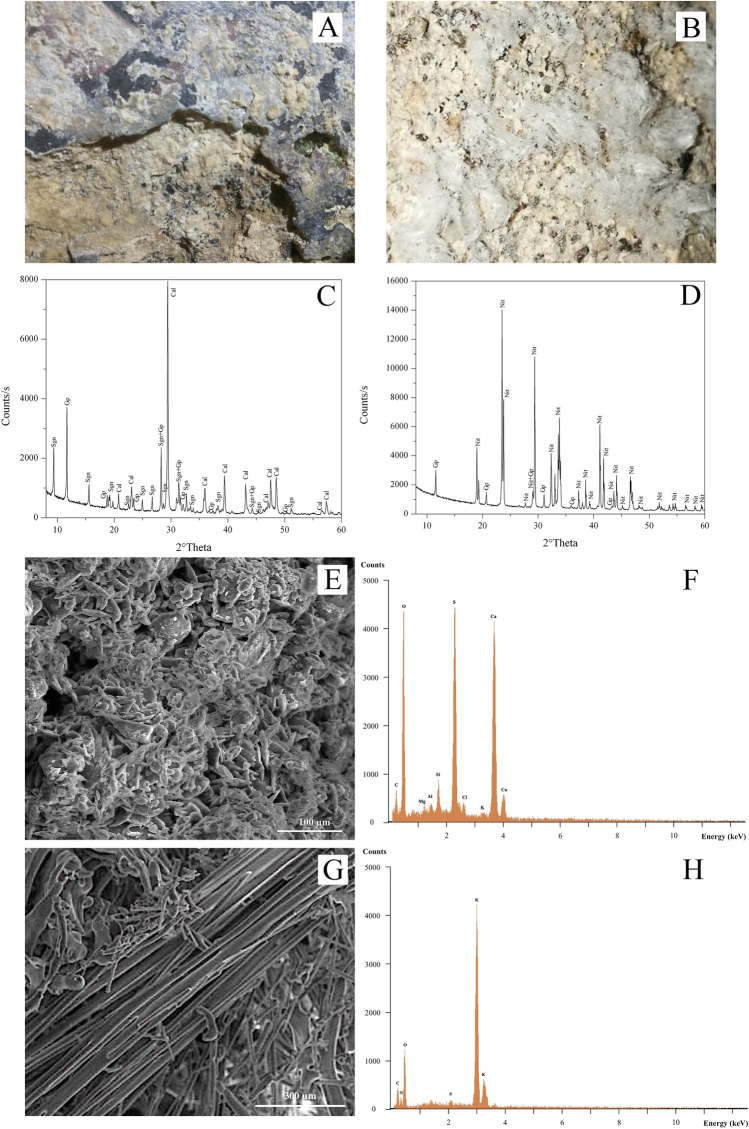
(A) Macroscopic image of the encrustating deposits and (B) efflorescence; (C) XRPD pattern of the encrustation and (D) of the efflorescence; (E) SE imaging of gypsum and (F) the corresponding EDS spectrum; (G) SE imaging of KNO3 whisker crystals and (H) the corresponding EDS spectrum. Legend for XRPD patterns: Sng = syngenite, Gp = gypsum, Cal = calcite, Nit = potassium nitrate (saltpeter)

Encrustations, which overall make up most of the degradation products (about 95%), are widespread in all the decorated walls. They show a whitish colour, thickness ranging from 1 to 5 mm, and an often botryoidal morphology (Fig. 6A). Efflorescences, on the other hand, are much less common and found only in the lower part of the north wall close to the panel of the *Madonna del Latte*, about 1 m above the ground as aggregates of white fibrous crystals (Fig. 6B).

The XRPD analysis revealed the mineralogical compositions of the encrustations and efflorescences. The former consist of gypsum, CaSO_4_.2H_2_O, and much smaller quantities of syngenite K_2_Ca(SO_4_).2H_2_O, while the efflorescences are composed of potassium nitrate (saltpeter) (Fig. 6C–D). The SEM images clearly highlight aggregates of lamellar gypsum crystals and the EDS spectra obtained from these phases allowed their mineralogical compositions to be verified (Fig. 6E–F). The SEM observations of the efflorescences clearly show the small diameter (few microns) of the KNO_3_ “whisker” (Fig. 6G–H).

ESM 3 shows the results of the thermo-hygrometric monitoring carried out from March 2019 to December 2020. The weekly average values ​​obtained from approximately 17,500 point measurements (hourly recordings) are plotted in the graph. Regarding 2019, a similar trend can be appreciated between T and RH, whose curves are almost superimposed, with both exhibiting the highest values ​​in the summer period. The average temperatures were around 17 °C (with oscillations from 15 to 19 °C between winter and summer seasons), while the relative humidity was around 85% (ranging from 82 to 88%). For 2020, opposite trends can be observed for the two measured microclimate parameters, which could be interpreted as a consequence of the forced closure of the site due to the COVID-19 pandemic. In the same time interval (2019–2020), the average values of temperature and relative humidity measured in the microclimatic stations located in the same territory (data provided by the SIAS Sicilian Region Service) were around 12 °C (winter) and 27 °C (summer), with a RH of 85% (winter) and 65% (summer).

### Pigments

#### Portable X-ray fluorescence analysis

An in situ and non-destructive inspection of the wall paintings was carried out using p-XRF in order to identify the chromophore elements and obtain information about the composition of the painted layers. A total of 11 measurements were performed on selected areas of the wall painting representations. Six XRF spectra were acquired from the polyptych *Teoria dei Santi* (ESM 4A-B) and five from the *Madonna del Latte* (ESM 4C–D). The results of the XRF analysis with the chemical compositions related to the different colours are summarised in Table 1.

**Table 1:** Results of the in situ p-XRF investigation of the wall paintings of the Grotta del Crocifisso, the sample codes, measurement points, analysed colours, detected elements, and inferred pigments are reported; *tr*, traces

Sample	Measure point	Colour	Element	Pigment
*Madonna del Latte*				
PG01-ML	Madonna’s mantle	Red	Ca, Fe, P (tr), K (tr)	Red ochre
PG02-ML	Madonna’s mantle	Yellow	Ca, Fe, P (tr), S (tr)	Yellow ochre
PG03-ML	Madonna’s aureola	Yellow	Ca, Fe, P (tr)	Yellow ochre
PG04-ML	Child Jesus’s dress	Green	Ca, Fe, Mn (tr), P (tr), K (tr)	Yellow ochre mixed with blue
PG05-ML	Madonna’s mantle	Blue	Ca, Fe (tr), P (tr), K (tr)	Not identified
*Teoria dei Santi*				
PG01-TS	S. Elisabetta’s mantle	Red	Ca, Fe, P (tr), S (tr), K (tr)	Red ochre
PG02-TS	Madonna Odigidria’s aureola	Yellow	Ca, Fe, P (tr), S (tr)	Yellow ochre
PG03-TS	San Leonardo’s background	Blue	Ca, Fe (tr), P (tr), S (tr)	Not identified
PG04-TS	San Giovanni Battista’s hair	Brown	Ca, Fe, P (tr), S (tr)	Brown ochre
PG05-TS	San Nicola’s mantle	Red	Ca, Fe, P (tr), K (tr)	Red ochre
PG06-TS	San Nicola’s background	Blue	Ca, Fe, P (tr), S (tr)	Not identified

The examined colours were as follows: red, yellow, green, brown, and dark blue. In the *Madonna del Latte* pictorial representation, the red, yellow, and blue pigments were investigated: dark blue in the Madonna’s mantle, yellow in the Madonna’s aureole, and green in the Child Jesus’ dress, respectively. Regarding the *Teoria dei Santi* representation, the red pigment in the *S. Elisabetta*’s and *San Nicola*’s mantles, yellow in the *Madonna Odigidria’s aureola*, the brown *in San Giovanni* Battista’s hair, and the dark blue pigment in *San Leonardo*’s and *San Nicola*’s backgrounds were examined.

Calcium and iron were the most abundant elements characterising the pigments along with traces of P, S, and K. The trace quantities of P could be generically attributed to biological activity or to relatively more recent organic contamination. Small amounts of S and K can reasonably be ascribed to the occurrence of secondary alteration products (gypsum, potassium nitrate, and potassium sulphate) which formed through the centuries both on the painted areas and directly in the supporting plaster body. The presence of Ca can be attributed to the lime-based binder of the pictorial film, while Fe was identified as the main chromophore element in the red, yellow, brown, and green pigments; indeed, it was present in considerable quantities, together with traces of Mn. These latter elements notoriously characterise the “ochres” which are among the most widely used natural mineral pigments in wall paintings (Siddall 2018). On the contrary, according to the p-XRF measurements, no specific chromophore elements were highlighted in the case of the blue pigment, apart from negligible quantities of Fe and traces of P and K (ESM 4D). Additional diagnostic methodologies were thus considered for a more in-depth investigation and some representative micro-fragments of the dark blue pigment were collected for analysis by SEM-EDS, FTIR, and RS.

#### Microstratigraphy by reflected light microscopy and SEM-EDS

**Fig. 7:**
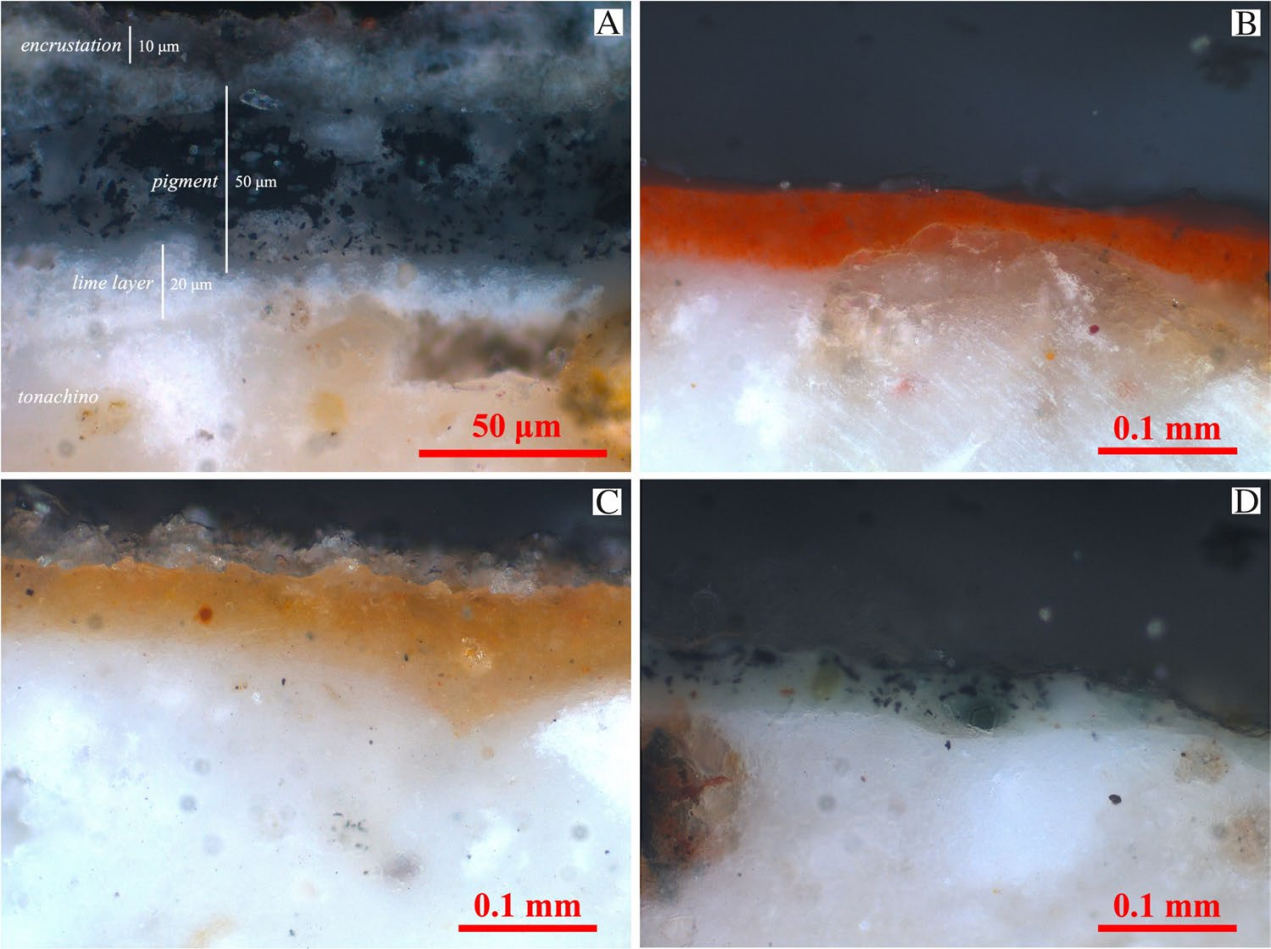
Representative micrographs of the cross-sections from the Grotta del Crocifisso wall paintings observed under reflected light. (A) Blue pigment, (B) red pigment, (C) yellow pigment, (D) green pigment. A and D are from the Madonna del Latte, while B and C are from the Teoria dei Santi pictorial representation

The observation of pictorial layers by reflected light microscopy performed on polished cross-sections allowed to highlight and comparatively evaluate the microstratigraphy and distribution of pigment particles. In this case study, the observed microstratigraphy has always revealed the presence of three different layers from the bottom to the top. A very thin, compressed, and smoothed pure lime layer (thickness less than 20 μm) can be observed over the *tonachino* layer. This latter is, in turn, covered by a top layer with relatively more concentrated pigment particles. Moreover, a secondary encrustation with variable thicknesses (10–40 μm) can also be observed (Fig. 7A).

The red and yellow pigment layers appear to be very dense, with thicknesses ranging from 20 to 50 μm for the red and from 50 to 100 μm for the yellow pigment. They both comprise very fine (mainly submicrometric) iron oxide particles, which are homogeneously distributed in the carbonated lime binder (Fig. 7B–C). The thickness of the green and blue layers mainly ranges from 20 to 50 μm. They both show the odd presence of irregularly shaped particles with rather variable sizes (from a few microns up to about 20 μm), heterogeneously scattered in the binder (Fig. 7A and D). The green pigment seems to have been obtained by mixing the blue one with yellow ochre.

**Fig. 8:**
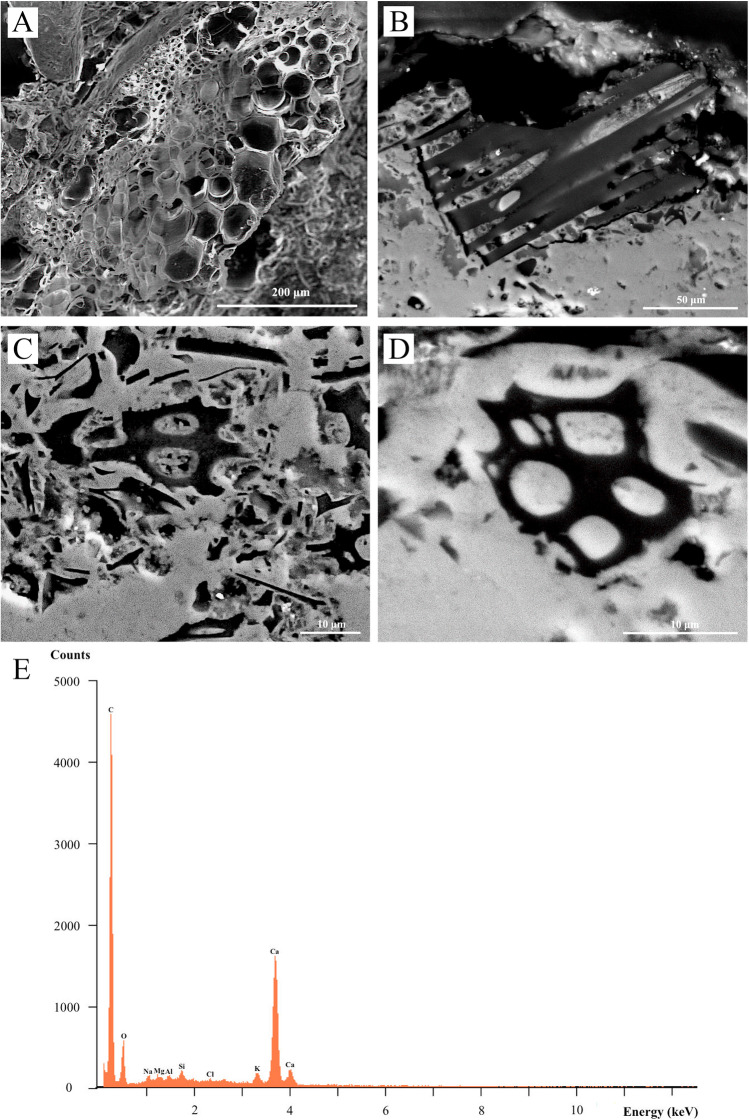
Scanning electron microscopy (SEM) investigation of the observed vegetal particles in the mural paintings from the Grotta del Crocifisso. (A) Secondary electron (SE) image showing a typical hexagonal, honeycomblike pattern; (B), (C), and (D) backscattered electron (BSE) image of the dispersed organic particles with varied morphologies; (E) EDS microanalysis spectra of the vegetal fragments

The reflected light microscopy of these dark blue-coloured micro-particles shows a peculiar morphology that may not be attributed to compounds of inorganic nature. The cross-section for the blue pigment was then observed using secondary electron imaging (SEI) to accurately emphasise the microstructure and morphology of the pigment micro-particles. This allowed an arrangement of hollow, hexagonal-shaped cells, characterised by thin steep vertical walls, resembling the typical honeycomb-like structure of plant leaves, to be highlighted (Fig. 8A). This result undoubtedly points to the presence of organic-derived material used for obtaining the blue pigment. Back-scattered electron imaging (BSEI) was then performed on polished cross-sections of the same representative samples for obtaining compositional information in order to verify the organic (vegetal) nature of the observed micro-particles as well as to highlight their microtextural features. The images shown in Fig. 8B, C, and D reveal particles of various sizes (few microns to tens of microns) and diversified morphologies ranging from tabular to lobate or elongated forms depending on their orientation. The greater fragments show round to ellipsoidal blobs with sharp outlines presumably representing the original hexagonal cavities, characterising the honeycomb pattern of the plant leaf, in-filled by the carbonated lime binder. By and large, the observed particles broadly appear as fragments of chopped plant leaves. Semi-quantitative EDS spot micro-analyses of the dark particles revealed high concentrations of carbon as well as notable amounts of Ca and minor peaks of K, Na, Mg, Al, Si, and Cl (Fig. 8E). No carbon sputtering was applied onto the sample when operating in low vacuum mode (ESEM) to avoid carbon interference with the EDS analysis. The presence of carbon as the most prominent chemical element thus demonstrates the genuine organic nature of the analysed particles. Accordingly, this evidence corroborates the observation made by optical microscopy and secondary electron imaging and strengthens the thesis on the use of an organic dye for the dark blue colour in the *Grotta del Crocifisso* wall paintings. Among the blue organic colourants, indigo has certainly one of the most used since remote antiquity (Cardon 2007; Aceto 2021). Therefore, all these observations lead us to strongly suspect that this latter may have been employed in the samples analysed in this case study. Hence, both FTIR and RS measurements were performed to confirm the above hypothesis.

#### FTIR and Raman spectroscopy

Table 2 provides the assignment of the most characteristic vibration bands (based on theoretical calculations from literature data) compared with the experimental ones obtained on the samples of the *Grotta del Crocifisso*. The FTIR measurements were ambiguous in highlighting the presence of indigo, which is mostly hidden by the stretching vibrations of calcite, (1409, ~ 875 and ~ 711 cm^−1^), gypsum (3534-3401, 1621, 671, 609 cm^−1^), and quartz (1140, 1119, 1012 cm^−1^), all representing major components of the lime binder (calcite), of the aggregate (quartz) and of the degradation products (gypsum). However, the vibrational stretching of the C–H group at ~ 2851 and ~ 2920 cm^−1^ strongly supports the presence of organic matter mixed with the aforementioned inorganic phases (ESM 5A).

**Table 2.** IR and Raman band assignment of the analysed blue pigment from the Grotta del Crocifisso

Band assignment	Calcite	Gypsum	Silicates/Quartz	Indigo
IR data	1450–1420, 890–870, 720–700	3547-3404, 1621, 1141-1128-1116, 669, 601	1085, 800, 780, 695, 514, 462	1626-1614-1583, 1482-1460-1410, 1319-1300, 1066-1022, 871, 751, 709, 698
Blue sample	3534, 3401, 2922, 2852, 2504, 1795, 1626-1621, 1440-1406, 1109, 1082, 1012, 871, 856, 823, 712, 666, 600			
Raman data (532 nm)	154, 278, 710, 1086	181, 415, 493, 617, 670, 1008, 1134	128, 206, 264, 354, 390, 450, 464, 697, 796, 808, 1069, 1162	248, 280, 547, 599, 670, 755, 858, 937, 1009, 1096, 1145, 1249, 1310, 1362, 1460, 1483, 1572, 1584, 1606
Blue sample	1695, 1596, 1439, 1352, 1220, 1084, 957, 904, 804, 636, 582, 571, 509			

The results of Raman spectroscopy are presented in ESM 5B where they are compared to the most recent indigo spectrum data available in the literature (Caggiani et al. 2016). An acceptable correspondence was found in this case, also considering that while the spectrum obtained from the literature was acquired on pure indigo, the same pigment was in the past normally applied together with organic binders such as beeswax, red or white eggs, or Sandarac resin (Manfredi et al. 2017). The indigo pigment, as revealed by the 532 nm laser with Raman peaks at 571, 1352, 1439, and 1596 cm^−1^, was thus considered compatible with the compositions reported in the literature (248, 547, 599, 1310, 1572, and 1584 cm^−1^), even if the presence of organic materials generates emission effects (fluorescence) which could interfere with the weaker Raman peaks emitted by the studied sample.

## Discussion

The results illustrated in the previous paragraphs, obtained using a multi-analytical approach, shed light in a systematic way on the whole “production process” that led to the creation of the decorative complex of the *Grotta del Crocifisso*. At first glance, it can be stated that in terms of selection criteria of the raw materials, procedures, and methods for the realisation of the supporting plaster and the pictorial films, the workers adapted to what was available locally thereby significantly reducing the production costs, but nonetheless demonstrating good technical skills.

The calcarenite outcrop where the hypogeum site has been carved (probably into a pre-existing underground stone quarry) shows a characteristic uniqueness, consisting of a detrital component of volcanic nature which reflects the geological evolution of the Hyblean territory in the Plio-Pleistocene. Its pore-size distribution is characterised by an almost bimodal trend, making this calcareous sandstone an “ideal substrate” for a plaster layer with the *arriccio* function. In fact, the capillary rise of water from the subsoil could not be over-hindered by any surfaces of discontinuity between natural stone material (calcarenite) and artificial stone material (plaster). Consequently, the precipitation of soluble salts on the external surface in the form of (easily removable) efflorescences would be reinforced, and, in the meantime, the risk of formation of dangerous sub-efflorescences would be relatively reduced (Randazzo et al. 2020).

The plaster used to prepare the wall to be frescoed was formulated in a conscious and optimal way, according to the available resources. The layer adhering to the natural stone (*arriccio*) is technically classifiable as an “artificial hydraulic mortar”, obtained by mixing an aggregate consisting of local, highly reactive volcanic products (submarine lavas and hyaloclastite) and an aerial lime. The binder, obtained through the “pozzolanic reaction” between lime and glassy volcanic lithoclasts in the presence of water, was applied to a particularly humid underground context such as the one studied. The presence of *cocciopesto* strengthens the hydraulic characteristics of the pinkish *arriccio*. The *tonachino* layer was designed differently by mixing local calcareous sand with an aerial lime-based binder. As a result of complete carbonation, the pigments were thus fixed more efficiently. These technological choices, on the whole, allow us to infer a certain control in the optimal use of the locally available raw materials.

Despite the selection of optimal raw materials and a wise use of technological knowledge, the extremely aggressive microclimate has inevitably led the wall painting to deteriorate over the centuries. In fact, as it was possible to appreciate from the thermo-hygrometer measurements, ​​while the average indoor temperatures fell in the optimal range 10–24 °C that is considered ideal for an appropriate conservation of wall paintings and frescoes, and the relative humidity values were always higher than the 55–65% threshold (after UNI 10829: 1999). Accordingly, from the calculation of the dew point, values ​dangerously close to the condensation conditions could be obtained, for both winter and summer periods. Overall, these trends contribute to our understanding of the deterioration forms detected in the *Grotta del Crocifisso* which outlined the urgent need for planning appropriate restoration interventions.

Among the identified alteration and degradation products, sulphates predominate, specifically gypsum and syngenite. The authigenic precipitation of gypsum on carbonate stone is due to the chemical reaction between sulphate (SO_4_^2-^) and calcite (CaCO_3_), with various possible origins of the sulphate anion from anthropogenic sources, marine aerosols, and also by volcanic emissions since the Etna volcano is only about 50 km from Lentini (Aroskay et al. 2021 and references therein). On the other hand, syngenite originates on calcareous substrate through the hydrolytic mobilisation of alkaline metals from volcanic glass in the presence of percolating solutions enriched in sulphate anions. This mechanism has already been observed in the “black crusts” developed on the monumental buildings of the historical centre of Catania (Alaimo et al. 1995). Regarding the salt efflorescences, the presence of saltpeter can be interpreted as being directly related to anthropogenic activities. In fact, the *Grotta del Crocifisso* was also used for a long time as a burial place by the Capuchin monks who lived in the nearby convent in the sixteenth and seventeenth centuries. Indeed, the adjacent *Grotta di Santa Lucia* was used as a nitrary, like several other places in Calabria and Sicily during the eighteenth century, to produce the nitrates necessary for the manufacture of gunpowder (Fasano 1787).

The multi-analytical approach (p-XRF, optical microscopy, SEM-EDS) revealed the presence of ochres in the red, yellow, brown, and green pigments. These inorganic geomaterials are easily available in the territory neighbouring the *Grotta del Crocifisso*. In fact, iron-bearing weathering lenses are quite common in the Hyblean region and have originated from the atmospheric oxidation of the Quaternary submarine volcanic products (Grasso et al. 1987; Carbone 2011). In the same territory, ochres may also be present in the residual deposits resulting from the dissolution of the exposed carbonate rocks. In this case study, the red and yellow ochres were mostly applied using the “fresco” technique (i.e. colours were applied on plaster that was not yet dry), in accordance with the autoptic investigations made by the restorers and the reflected light microscopy observations. Some pigments were also applied with “mezzo fresco” technique, particularly regarding the darker ones, which were used to emphasise the contours and folds of clothing. This statement is indirectly corroborated by the relatively stronger degradation observed in these specific parts of the pictorial representations as the pigment was not perfectly incorporated within the binder. It is well known from past literature that a wall painting made entirely with the “fresco” technique is rather rare and that painters were often experimenting and applying colours even when the plaster was completely dry (Casadio et al. 2004). Moreover, the painters of the Middle Ages applied colours while looking for requirements such as brightness, intensity, and saturation, without shades and halftones, to bring out the expressive power of the figure and its symbolic meaning. In this case, the colour palette reflects these criteria being composed of yellow, brown, red, and green ochres as well as an organic blue pigment, mixed with *bianco di San Giovanni* (also called “lime white”).

The blue pigment was used in several paintings of the *Grotta del Crocifisso*. Since the Middle Ages, the blue colour has been used to symbolise spirituality and transcendence, being generally made with the dye obtained from lapis lazuli (or azurite) if economic resources allowed it. However, our experimental data demonstrated that the blue pigment used on the Madonna’s mantle and on other studied figures has an organic nature. In fact, the p-XRF and SEM-EDS analyses did not show the presence of chromophore metals, and, on the contrary, they showed the predominance of carbon (Fig. 8E). Thus, mainly by means of Raman spectrometry, it was possible to identify woad, a pigment extracted from the plant *Isatis tinctoria*. This latter is a biennial herbaceous plant of the *Brassicaceae* family, with a height ranging from 40 to 120 cm. It is a weed whose fresh leaves contain the precursors of the indigo pigment, obtained through a maceration system prior to being dried and powdered (Guarino et al. 2000). Woad was also known to the Romans and was widely used throughout Europe and in central-southern Italy primarily as a textile dye and, only secondarily, as a pigment for fine arts. The extraction and dyeing were in fact quite complicated processes, so the indigo pigment was very precious and appreciated by the nobility. Its use grew exponentially until the first half of the seventeenth century, when the advent of the cheaper indigo traded from the Indies (*Indigo tinctoria*, already in powder form) strongly supplanted woad cultivation in Europe, except for southern Italy, where the cultivation of woad increased from the eighteenth century (Guarino et al. 2000).

Indigo precursors can be extracted from the leaves of *Isatis tinctoria* through an extraction process that involves the enzymatic hydrolysis of heterocyclic aromatic compounds (Stoker et al. 1998a), mainly, Isatan B (indoxyl-5-ketogluconate) and, to a lesser extent, Indican (indoxyl-β-D-glucoside). Isatan B and Indican are initially hydrolysed through a reaction of catalysis by the enzyme b-glucosidase contained in the leaves of the same plant. The reaction normally proceeds at a weakly acidic pH, between 4.8 and 6.0 (Kiernan 2007). This mechanism leads to the formation of indoxyl molecules: a water-soluble yellow-green solid. Under oxidising conditions and in an alkaline environment, the reaction proceeds up to the dimerisation of the indoxyl (with a prevalence of the enol form) and the consequent formation of insoluble indigo (ESM 6).

For a simple comparative experimental test, the pigment was obtained in the laboratory in order to compare it with what was detected instrumentally in the *Grotta del Crocifisso* (ESM 7). A proper quantity of *Isatis tinctoria* leaves (about 5 kg), grown spontaneously in the surrounding area, was sampled. The leaves were placed in HPDE (high-density polyethylene) containers, filled with a HCl 10% (v/v) aqueous solution to undergo maceration (after Stoker et al. 1998b). The containers were then placed in an environment devoid of light at a controlled temperature of 22 °C for around 36 hours. The obtained maceration water was thus filtered with a standard US steel Mesh 10 (2 mm). Subsequently, Ca(OH)_2_ was gradually added to the filtered extract until a pH of 9.5 was reached. Air was blown into the previously filtered and alkalised maceration water for a couple of hours by using a vacuum pump (oxidation step). The filtered solid residue was placed in a ventilated oven at 40 °C. As soon as the obtained residue had been completely lyophilised, a few drops of concentrated HCl diluted with water were added until the final blue colour was obtained. Finally, the pigment was left decanting and then recovered with the aid of filter paper and dried at room temperature. The obtained pigment was observed under a reflected light microscope and compared with the corresponding plaster samples. It is interesting to note how the comparison made by optical microscopy revealed the same morphological features of the pigment particles and a blue hue very similar to that found in the historical plaster, thus corroborating the results obtained through diagnostic investigations (ESM 7).

The attestation of indigo/woad blue in the wall paintings of the *Grotta del Crocifisso* represents an interesting result of this paper. In fact, the use of this pigment in the specialised literature is considered suitable mainly for watercolours and tempera paintings, since it would seem to be incompatible with very alkaline mediums such as those of fresco paintings (Schweppe 1997; Aceto 2021). This common opinion, which was already explicitly expressed in Cennino Cennini’s *Libro dell’Arte* (chapter LXXII), is somehow corroborated by the fact that its identification in wall paintings is still relatively rare today. Among the most important examples in Italy are the *Last Supper* by Leonardo da Vinci (Monastery of Santa Maria delle Grazie, Milan), Crypt of *Santo Stefano* (Lecce), *Santa Maria della Steccata* (Parma), and frescoes on the *Legend of the True Cross* by Piero della Francesca in Arezzo (Schweppe 1997; Bersani et al. 2004; Fico et al. 2016; Aceto 2021). The present report is the first recovering of this pigment in medieval Sicily and deserves proper attention, as, from ongoing research, it could also be present in several other similar Sicilian contexts. The blue pigment in the *Grotta del Crocifisso* was applied with “mezzo fresco” technique and therefore under drier conditions than the other pigments that were applied with the “fresco” technique. In fact, since woad is relatively unstable under strongly alkaline pH conditions, it was consciously applied when the plaster surface was already dry and almost completely carbonated, thus promoting its stability.

## Conclusions

The investigation of the wall paintings from the *Grotta del Crocifisso* in Lentini required the use of various instrumental methodologies to better understand the painting techniques used as well as to provide the necessary indications for carrying out restoration works aimed at eliminating the deterioration products and improving the legibility of this work of art.

The set of analyses carried out on the studied wall paintings provided a detailed characterisation of all the constituent materials (stone substrate, plasters, pigments) as well as the forms of alteration and degradation. These data are firstly highly useful for the planning of the restoration and recovery interventions required to reaffirm the importance of this monumental complex in the Sicilian territory. The investigations also yielded significant information on the pigments used and highlighted the peculiar presence of indigo/woad blue, the use of which in past years has always been likely underestimated by diagnostic approaches. A detailed understanding of its application and the medium used to paint it require further research, but the studies carried out provide a good basis for future knowledge of the artistic techniques of wall paintings in the Hyblean territory, in Sicily, and in the whole region of central-southern Italy, especially between the thirteenth and sixteenth centuries.

## Supplementary Information

ESM 1 (DOCX 14859 kb)
