# Supplementary material for: Multi-analytical study of the medieval wall paintings from the rupestrian church Grotta del Crocifisso at Lentini (eastern Sicily): new evidence of the use of woad (Isatis tinctoria)
Source: Archaeol Anthropol Sci. 2022 Sep 3;14(9):183. doi: 10.1007/s12520-022-01656-6 (PMC9440324; doi:10.1007/s12520-022-01656-6)

**Multi-analytical study of the Medieval wall paintings from the rupestrian church *Grotta del Crocifisso* at Lentini (eastern Sicily): new evidence of the use of woad (*Isatis tinctoria*).**

Montana G.^1^, Giarrusso R.^2†^, D’Amico R. ^3^, Di Natale B.^4^,

Vizzini M.A.^2^, Ilardi V.^5^, Mulone A.^2^, Randazzo L.^1*^, Ventura Bordenca C.^1^

*^1^ Dipartimento di Scienze della Terra e del Mare (DiSTeM) - Università degli Studi di Palermo, Italy*

*^2^ GEOLAB s.r.l Laboratorio di Ricerca e Sperimentazione sui Materiali - Via de Spuches – Carini (Palermo), Italy*

*^3^Conservator, Parco Archeologico di Leontinoi (Regione Siciliana), Lentini (Siracusa), Italy*

*^4^Professional Restorer, Palermo, Italy*

*^5^Dipartimento di Scienze e Tecnologie Biologiche Chimiche e Farmaceutiche (STEBICEF), Università degli Studi di Palermo (Italy)*

*^*^Corresponding author:* [*luciana.randazzo@unipa.it*](mailto:luciana.randazzo@unipa.it) *(Randazzo L.)*

^†^*Deceased January 13, 2022*

**ESM1.** Sample description and analytical methods. **OM** = optical microscopy; **XRPD** = X-ray powder diffraction; **SEM-EDS** = scanning electron microscopy using energy dispersive spectroscopy; **FTIR** = Fourier-transform infrared spectroscopy; **RS** = Raman spectroscopy.

| **Sample Code** | **Sample description** | **Collection Point** | **Analytical Methods** | | | | | |
| --- | --- | --- | --- | --- | --- | --- | --- | --- |
|  |  |  | OM | XRPD | MIP | SEM-EDS | FTIR | RS |
| **Local calcarenite** |  |  |  |  |  |  |  |  |
| CC-1 | *lithic substrate (calcarenite)* | *entrance wall* | x | x | x | - | - | - |
| **Plasters** |  |  |  |  |  |  |  |  |
| CR/AR-1 | *arriccio (grayish rendering layer)* | *Teoria dei Santi* (*San Nicola*, lower part) | x | - | x | x | - | - |
| CR/AR-2 | *arriccio (pinkish rendering layer)* | *Madonna del Latte* (right middle part) | x | - | x | x | - | - |
| CR/RN-1 | *arriccio (pinkish rendering layer)* | *Madonna del Latte* (lower right) | x | - | x | x | - | - |
| CR/RN-2 | *arriccio (grayish rendering layer)* | *Teoria dei Santi* (*San Giovanni Battista*, lower right) | x | - | x | x | - | - |
| CR/FC-1 | *tonachino (finishing layer)* | *Madonna del Latte* (lower right) | x | - | x | x | - | - |
| CR/FC-2 | *tonachino (finishing layer)* | *Teoria dei Santi* (*San Nicola*, lower part) | x | - | x | x | - | - |
| **Degradation** |  |  |  |  |  |  |  |  |
| ENC-1 | concretionary deposits | *Teoria dei Santi* (*San Giovanni Battista*, lower part) | x | x | - | x | - | - |
| ENC-2 | concretionary deposits | *Teoria di Santi* (*San Nicola*, lower part) | x | x | - | x | - | - |
| ENC-3 | concretionary deposits | *Teoria dei Santi* (*San Nicola*, lower part) | x | x | - | x | - | - |
| EFF-1 | fibrous crystal aggregate | lower part of the north wall (close to *Madonna del Latte* panel) | - | x | - | x | - | - |
| **Pigments** |  |  |  |  |  |  |  |  |
| PG01-ML | Red | *Madonna del Latte* | x | - | - | x | - | - |
| PG02-ML | Yellow | *Madonna del Latte* | x | - | - | x | - | - |
| PG03-ML | Yellow | *Madonna del Latte* | x | - | - | x | - | - |
| PG04-ML | Green | *Madonna del Latte* | x | - | - | x | - | - |
| PG05-ML | Blue | *Madonna del Latte* | x | - | - | x | x | x |
| PG01-TS | Red | *Teoria dei Santi* | x | - | - | x | - | - |
| PG02-TS | Yellow | *Teoria dei Santi* | x | - | - | x | - | - |
| PG03-TS | Blue | *Teoria dei Santi* | x | - | - | x | x | x |
| PG04-TS | Brown | *Teoria dei Santi* | x | - | - | x | - | - |
| PG05-TS | Red | *Teoria dei Santi* | x | - | - | x | - | - |
| PG06-TS | Blue | *Teoria dei Santi* | x | - | - | x | x | x |

**ESM2.** (A) Plan relief of the underground monumental complex of the *Grotta del Crocifisso*; (B) panel of the *Teoria dei Santi* before the restoration interventions, extensively affected by the effects of alteration and degradation (encrustations, efflorescences, sub-efflorescences, intergranular decohesion, detachments).


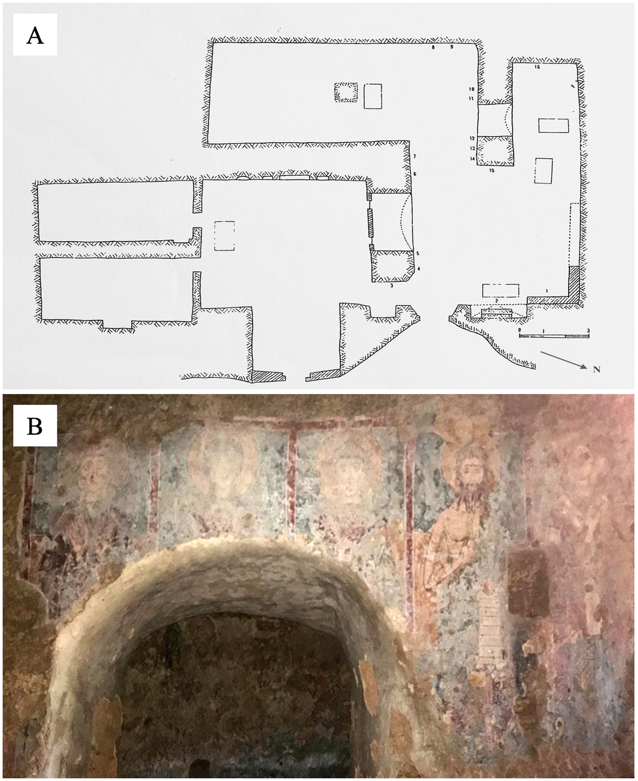


**ESM3.** Annual variation of temperatures (T°C) and relative humidity (RH%) in the *Grotta del Crocifisso.*


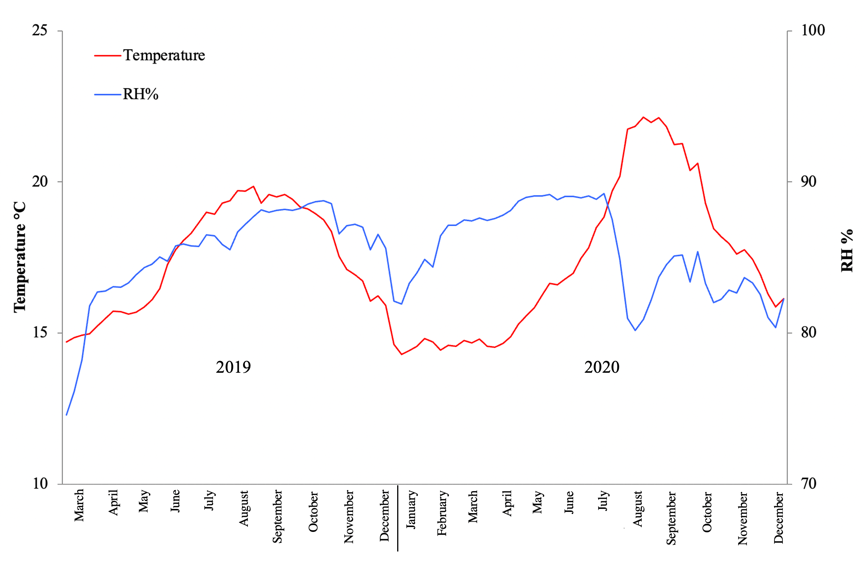


**ESM4.** Some representative examples of the in situ p-XRF analysis performed on the wall paintings of the *Grotta del Crocifisso* showing measurement points (left) together with their corresponding XRF spectra (right). (A) Red pigment of the *S. Elisabetta*’s mantle (sample PG01-TS); (B) yellow pigment of the *Madonna Odigidria*’s aureola (sample PG02-TS); (C) green pigment of the Child Jesus’s dress (sample PG04-ML); (D) blue pigment of the *Madonna*’s mantle (sample PG05-ML). The bright points in the images represent the beam spots of the XRF analyses.


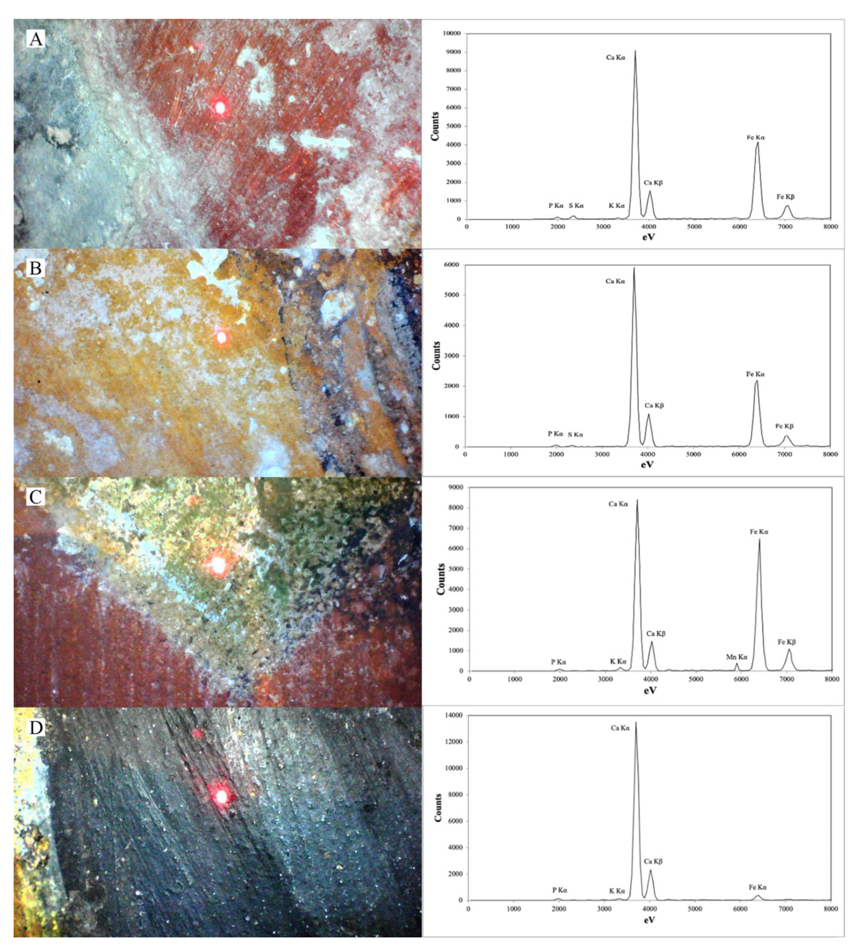


**ESM5.** FTIR (A) and Raman (B) spectra of the dark blue pigment from the *Grotta del Crocifisso*.


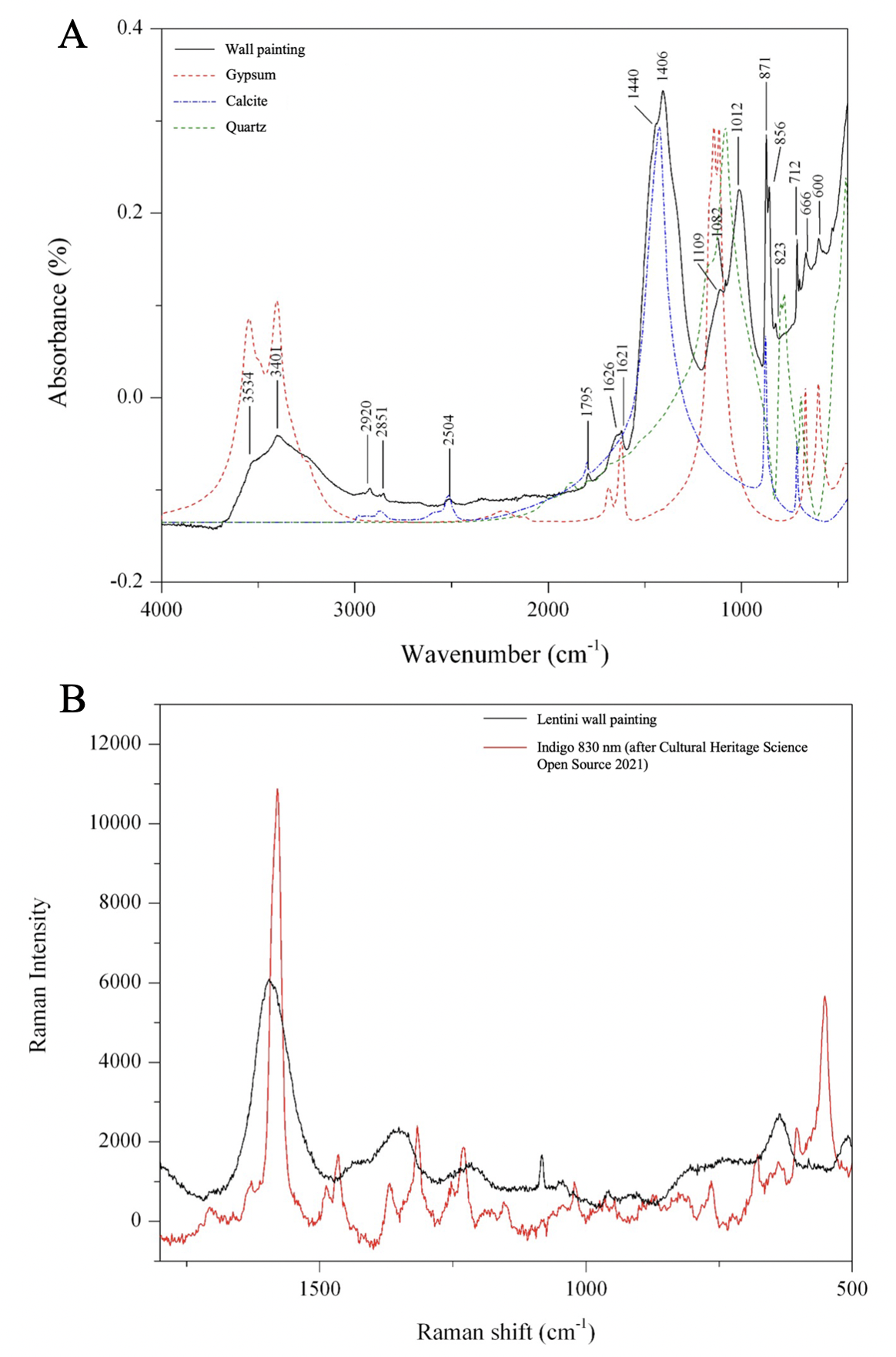


**ESM6.** Formation mechanism of blue indigo from Isatan B and Indican precursors under oxidising conditions.


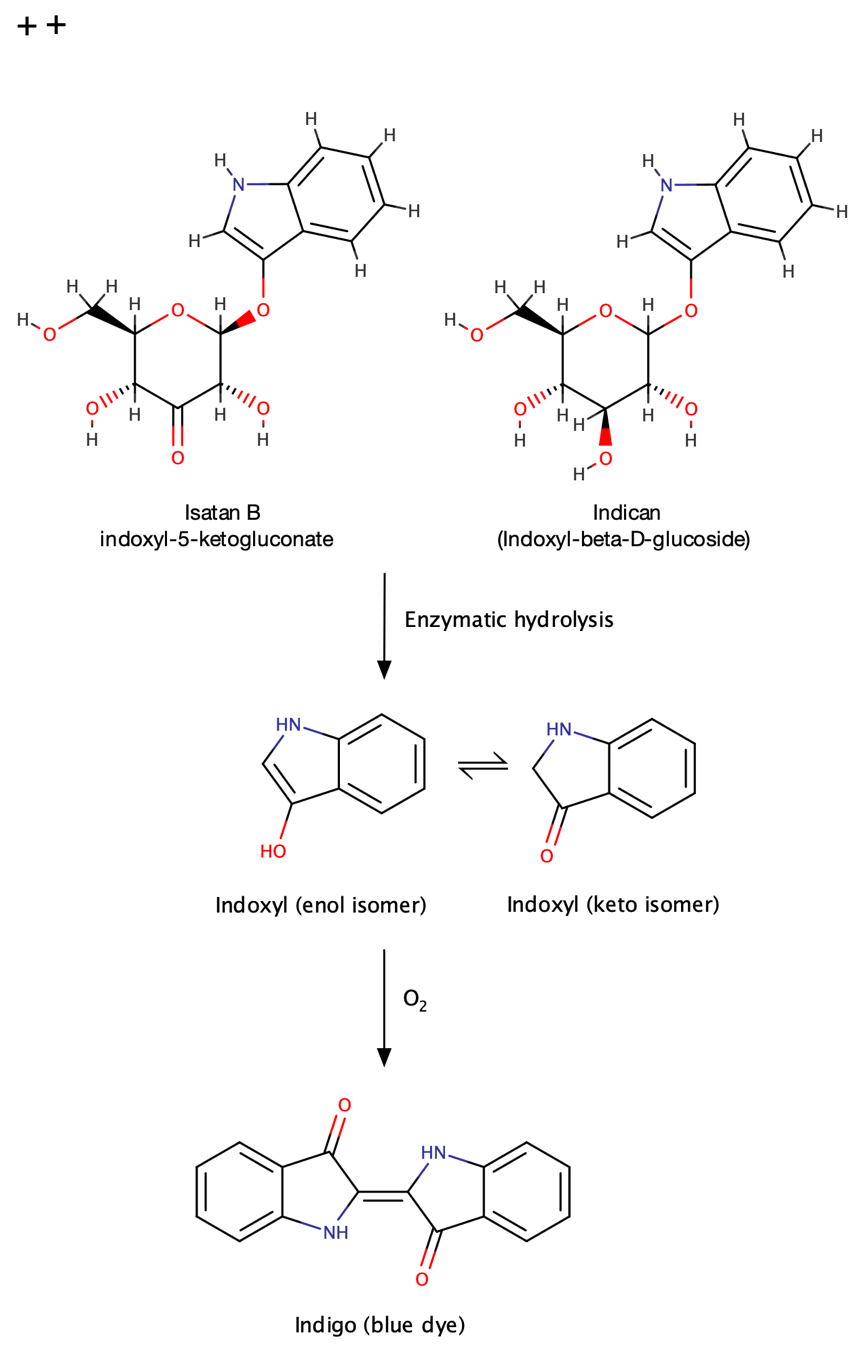


**ESM7.** Experimental cycle for the extraction of the blue pigment. 1: Sampling of the plant; 2: maceration of the plant leaves in a HCl 10% (v/v) aqueous solution; 3: filtering of the maceration water and addition of hydrated lime powder until reaching a pH of 9.5; 4: air insufflation of the alkalised maceration water with a vacuum pump (oxidation step); 5 and 6: addition of concentrated HCl diluted with water until the formation of the blue colour. A morphological comparison under reflected light microscopy between the blue pigment in the samples collected from the *Grotta del Crocifisso* (A and B) with that obtained experimentally in the laboratory (C and D) is shown in the lower right of the image.


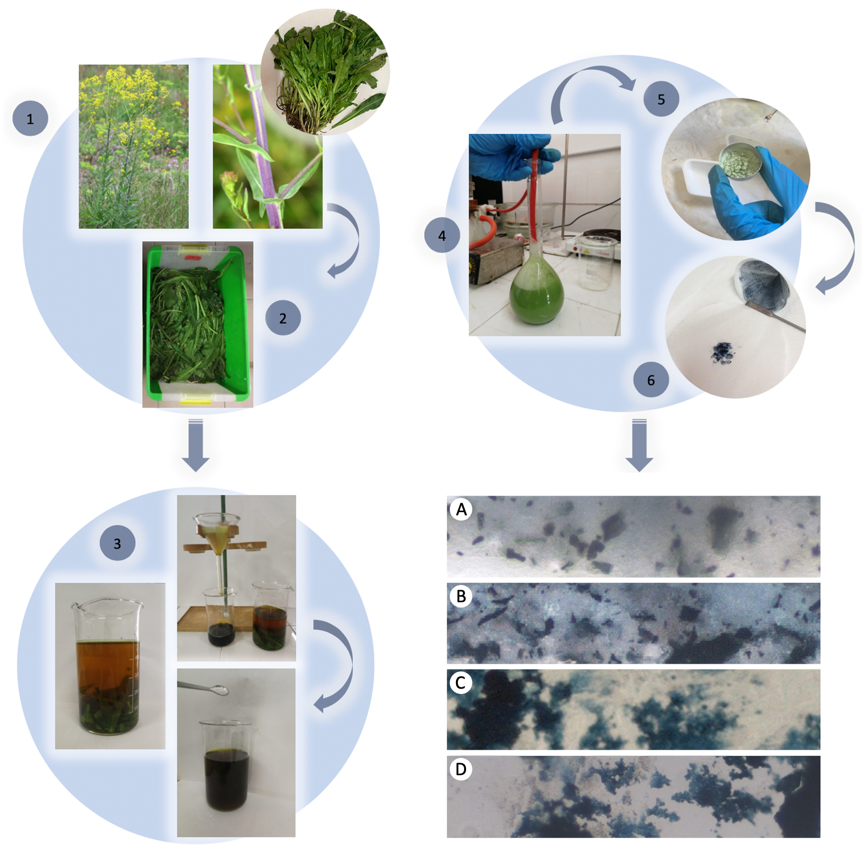

Supplement: Supplementary file 1 — (DOCX 14859 kb) [file 12520_2022_1656_MOESM1_ESM.docx]
